# Dataset on the Hanoian suburbanites’ perception and mitigation strategies towards air pollution

**DOI:** 10.1016/j.dib.2020.106414

**Published:** 2020-10-19

**Authors:** Quy Van Khuc, Tri Vu Phu, Phuong Luu

**Affiliations:** a Faculty of Economics and Business, Phenikaa University, Hanoi 12116, Vietnam; b Department of Economics, National Economic University, Hanoi 100000, Vietnam

**Keywords:** Air pollution, Perception, Solutions, Environment policy, Vietnam

## Abstract

Although air pollution has become a significant global environmental problem posing many negative impacts on human health and society, there has been a little known about how people think and respond to it, especially in developing and emerging countries. This article presents a dataset on how the suburbanites perceived and reacted with air pollution in Vietnam, an emerging country in Southeast Asia. A stratified random sampling technique and a face-to-face interview method were employed to survey 302 inhabitants living within Hanoi suburban, during November and December 2019. The constructed data contains three groups of information: (1) perspectives on air quality, causes, and impacts of polluted air, (2) respondent's preventative measures to air pollution, and suggested solutions to improve air quality, and (3) demographic information of interviewees. The dataset could be useful for many scholars who want to conduct a further in-depth study and or environmentalists, policymakers who want to keep society informed about the air pollution-related progression, which could help design a desirable policy for mitigating and or controlling air pollution in Vietnam and beyond.

## Specifications Table

**Table:** 

Subject	Physical Sciences, Social Sciences
Specific subject area	Environmental Science, Environment management
Type of data	Table Figures Excel files
How data were Acquired	Data were collected using a field survey. A questionnaire-based face-to-face interview method was used to survey inhabitants during November and December 2019. Data converted into .xlsx format for formal analysis in Stata version 11.0
Data format	Raw Analyzed
Parameters for data collection	The target population of the survey was inhabitants who live in seven out of eight suburban districts in Hanoi, including Ha Dong, Cau Giay, Tay Ho, Bac Tu Liem, Nam Tu Liem, Thanh Xuan, and Hoang Mai.
Description of data collection	The data was conducted through a field survey in Hanoi using a stratified random sampling technique
Data source location	Information was collected from Hanoi (Latitude 21° 1′ 42″ N, Longitude 105° 51′ 12″ E), Vietnam
Data accessibility	Repository name: Mendeley repository Data identification number: DOI: 10.17632/rbh7nksbtc.1 Direct URL to data: http://dx.doi.org/10.17632/rbh7nksbtc.1

## Value of the Data

• The dataset will be useful for researchers who want to learn the perception and the mitigation practices of the urban citizens towards air pollution
• The dataset will be helpful for researchers who wish to conduct comparative studies on air pollution in Hanoi, Vietnam, and different cities or different countries in the world.
• The constructed dataset will be useful for the environmentalists and policymakers who want to seek science-based solutions and or design the appropriate policies to mitigate the negative impacts of air pollution.

## 1 Data Description

During many consecutive months in late 2019, due to the high value of air quality index (AQI), Hanoi was ranked as one of the most polluted capital cities globally [1,2]. Under this context, this study was conducted to learn about and or examine suburbanites’ perception and mitigation strategies for reducing air pollution. From November to December 2019, a total of 302 inhabitants who live within seven suburban districts in Hanoi was surveyed through a questionnaire consists of 40 items. After eliminating some incomplete and highly implausible answers, our raw data includes 290 observations and has information on (1) public knowledge and awareness regarding air pollution, and (2) the respondent's preventative measures and suggested solutions to improve air quality, and (3) demographic information of interviewees. The constructed dataset not only showed how well urban citizens perceived air pollution but also indicated how well they respond to mitigate and or control the impact of polluted air on health and society. The data can offer many insightful implications for a better environment policy in Vietnam and similar places in the world.

**Fig. 1:**
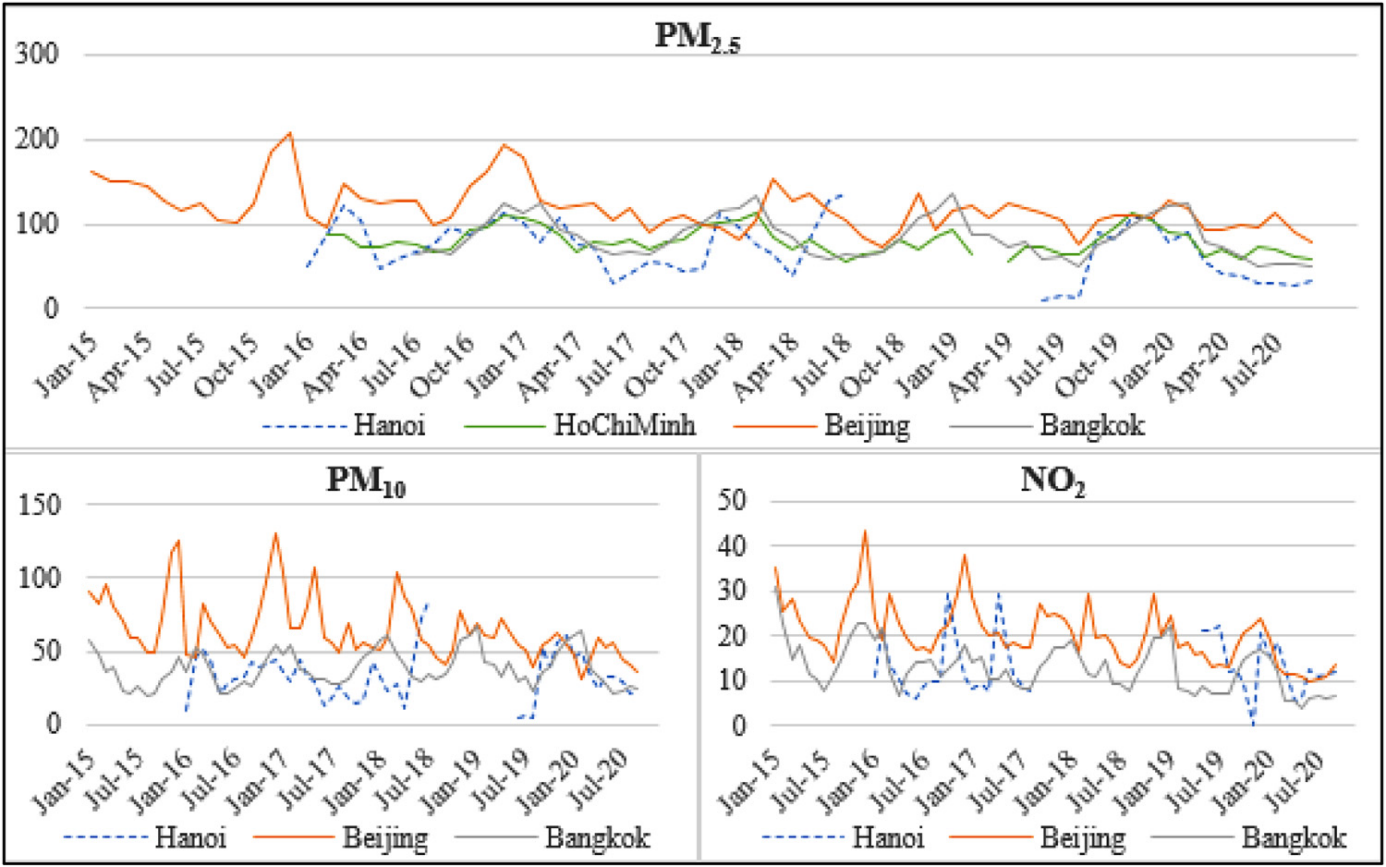
Air quality in Hanoi and some neighboring cities.

Sources: World Air Quality Index (WAQI.info). Data period: Jan 2015-Sep 2020.

**Air pollution in Hanoi and at district levels**. The citizen's perception toward air pollution might vary depending on the actual pollution levels in the living space. Therefore, it is necessary first to understand the quality of air in Hanoi and subsequently at the district level. Fig. 1 gives a rough idea of how polluted the Hanoi's atmosphere is. Due to data availability, we illustrate the air quality through three key indicators: the amount of the atmospheric particulate matter with a diameter of less than 2.5 micrometers (PM_2.5)_, the similar PM_10,_ and the concentration of nitrogen dioxide in the atmosphere. Starting from a PM_2.5_ level of 35.4 [1], the air is considered harmful to human life. Accordingly, Hanoi's air was mostly unhealthy on a majority of days in the 2016–2020 period. Overall, Hanoi's air pollution issue can be comparable to that in Bangkok, though it is slightly less severe than the problem in Beijing.

Since there are few air quality monitoring stations in Hanoi, it is quite challenging to obtain precise air quality information at the district level. Out of seven districts in our study, there is no air monitoring station in two districts (Ha Dong and Thanh Xuan). The recent air quality in the five remaining areas is exhibited in Fig. 2a. The indicator used in Fig. 2a is the Daily Air Quality Index (AQI) that measures the overall quality of air. The atmosphere with an AQI level of above 100 is considered polluted and has adverse impacts on human health.

**Fig. 2a:**
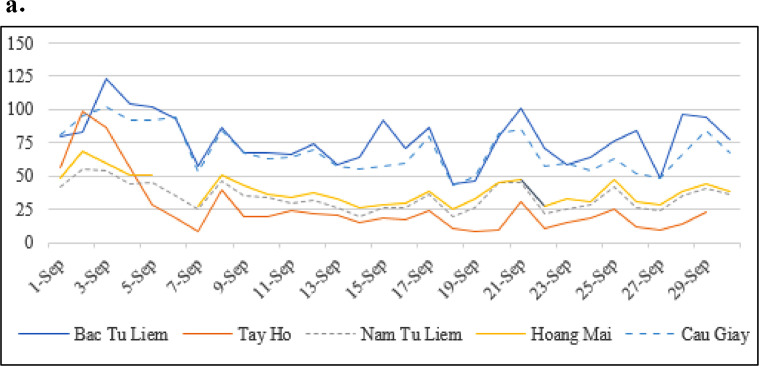
Air Quality Index in five suburban districts.

Sources: Center for Environment Monitoring (http://enviinfo.cem.gov.vn) and the United Nations International School Hanoi. Data of Cau Giay district is the average of two stations located within the area. Data period: September 2020.

Some specific districts have more extended historical data. Fig. 2b illustrates the air quality in three areas (Cau Giay, Nam Tu Liem, and Hoang Mai) through four key indicators of PM_2.5_, PM_10_, carbon monoxide (CO), and nitrogen dioxide (NO_2_).

**Fig. 2b:**
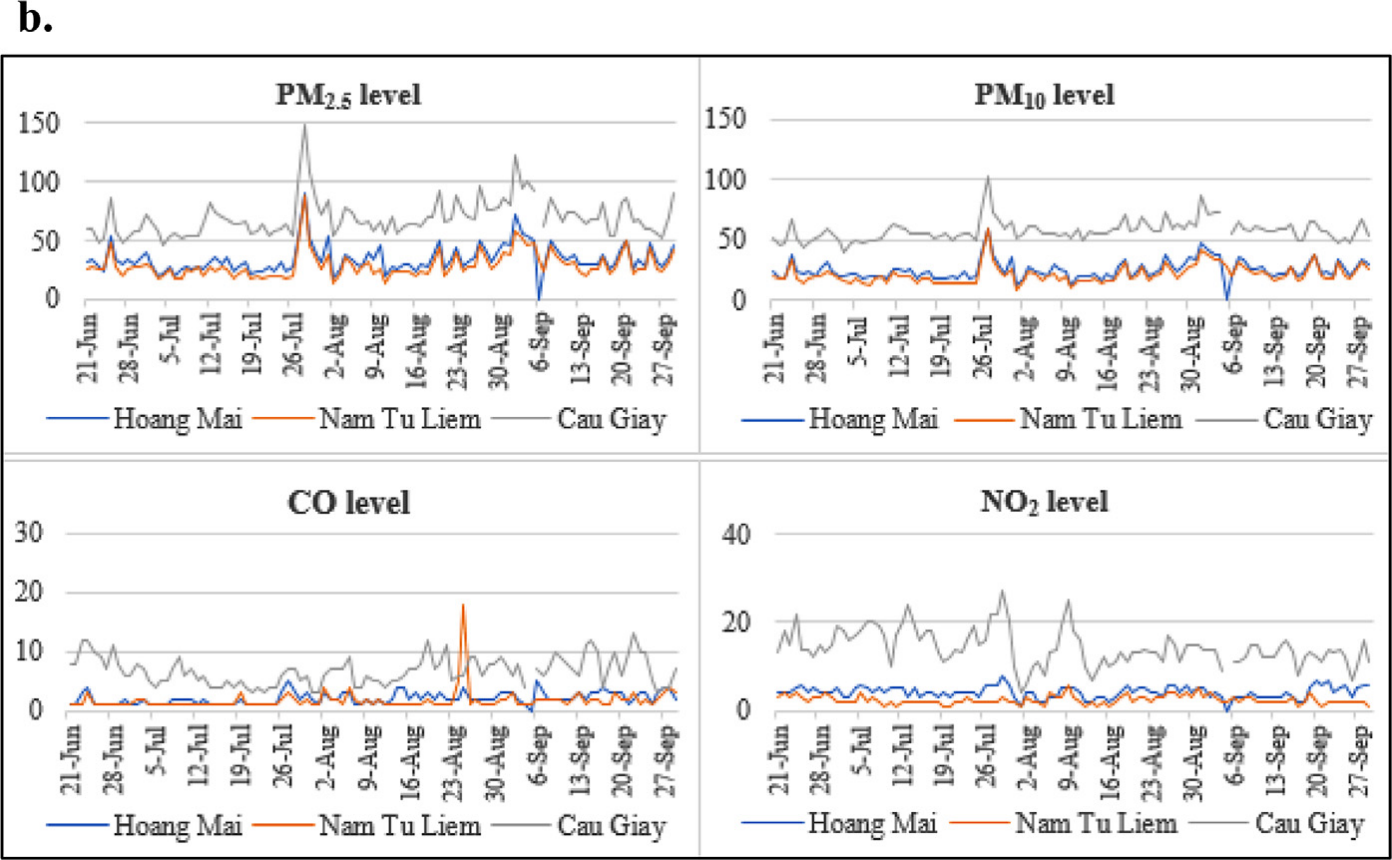
Air quality indicators of selected districts.

Sources: Center for Environment Monitoring. Data period: June 21, 2020 - September 29, 2020.

Overall, Cau Giay and Bac Tu Liem seem to have higher air pollution levels than the other three districts, namely Hoang Mai, Nam Tu Liem, and Tay Ho.

**Data descriptions**. The data description includes eight tables representing three main groups of information: perception, solutions, and demographical information of interviewees. Tables 1 and 2 show respondents’ understanding of air quality and the impacts of polluted air on their lives. Overall, most interviewees poorly evaluated the air quality in their living districts and the whole city. Many had a significantly low level of satisfaction with air quality and felt that the air is getting more and more polluted. The reduced air quality, with heavy smog and unpleasant smell (Table 2), has caused considerable concerns among interviewees and greatly impacted their daily lives.

**Table 1:** Perspectives on air quality and impacts of polluted air.

Dimensions	N	Mean	SD	SE	95% CI		Variable meaning and measurement
					Lower Bound	Upper Bound	
AirDistrict	290	3.66	0.82	0.05	3.56	3.75	Air quality of the living district. Measured on a five-point scale (1=very good; 2=good; 3=normal; 4=bad; 5=very bad)
AirVsOtherDistrict	282	0.85	0.36	0.02	0.81	0.89	Does air quality vary across districts? Measured on a binary scale (1= yes; 0=no)
AirCity	290	3.97	0.59	0.03	3.90	4.03	Air quality of Hanoi. Measured on a five-point scale (1=very good; 2=good; 3=normal; 4=bad; 5=very bad)
AirCityVsClosedCity	290	3.07	0.48	0.03	3.01	3.12	Air quality in Hanoi compared to neighboring cities. Measured on a four-point scale (1= better than; 2= same as; 3=worse than; 4= don't know)
AirCityVsSouthCity	290	3.04	0.95	0.06	2.93	3.15	Air quality in Hanoi compared to southern cities. Measured on a four-point scale (1= better than; 2= same as; 3=worse than; 4= don't know)
AirDistrictVsPast	288	2.86	0.43	0.03	2.81	2.91	Air quality of the living district compared to the past. Measured on a three-point scale (1=getting better; 2= unchanged; 3=getting worse)
AirCityVsPast	287	2.94	0.29	0.02	2.91	2.97	Air quality in Hanoi compared to the past. Measured on a three-point scale (1=getting better; 2= unchanged; 3=getting worse)
MorePolluted	290	0.97	0.18	0.01	0.94	0.99	Is Hanoi getting more and more polluted? Measured on a binary scale (1=yes; 0=no)
AirSatisfaction	287	2.02	0.82	0.05	1.93	2.12	Air satisfaction level. Measured on a five-point scale (1=very dissatisfied; 2= dissatisfied; 3=normal; 4=satisfied; 5=very satisfied)
AirCurrent	288	40.02	18.80	1.11	37.84	42.20	Evaluation of current air quality. Measured on a 0–100 scale
AirWish	289	81.12	13.05	0.77	79.61	82.63	Wish of air quality in the future. Measured on a 0–100 scale
PollutionConcern	290	1.86	0.85	0.05	1.76	1.96	Concerns regarding air pollution. Measured on a five-point scale (1=very concerned; 2=concerned; 3=normal; 4=not concerned; 5=do not care)
ImpactDegree	290	1.63	0.69	0.04	1.55	1.71	Impacts of polluted air. Measured on a four-point scale (1=very impacted; 2=impacted; 3=normal; 4=not impacted)
AirDisease	286	0.76	0.62	0.04	0.69	0.83	Disease caused by air pollution. Measured on a three-point scale (0=no; 1=yes; 2=don't know)

*Notes*: SD, SE, and CI stand for standard deviation, standard error, and confidence interval, respectively. For the more detailed information of dimensions, see the codebook at: http://dx.doi.org/10.17632/rbh7nksbtc.1.

**Table 2:** Uncomfortable things about air pollution.

Dimensions	Number	Percentages
Both heavy smog and unpleasant smell	182	62.8%
Heavy smog	95	32.8%
Unpleasant smell	9	3.1%
Others	4	1.4%

*Notes*: One interviewee can choose multiple causes.

Table 3 below demonstrates the response of interviewees to the causes of increasing air pollution. The finding shows that increased traffic and traffic congestion, construction, and population are mainly responsible for reduced air quality with each were agreed by 70–80% of all interviewees. The three are followed by smog from burned straws in the suburbs, an idiosyncratic cause of polluted air in Hanoi that is not present elsewhere in other big cities in Vietnam.

**Table 3:** Causes of increased air pollution.

Causes	Number	Percentages
Increased traffic and traffic congestion	235	81.0%
Increased construction (increased urbanization)	225	77.6%
Increased population/immigration	201	69.3%
Smog from burned straws in the suburbs of Hanoi	88	30.3%
Other causes (the use of honeycomb charcoal, consciousness, etc.)	35	12.1%
Climate change	23	7.9%
Air quality is not getting worse compared to the past	12	4.1%

*Notes*: Interviewees can choose multiple causes.

Table 4 illustrates the various impacts of polluted air. A vast majority of interviewed residents (92.1%) said that dirty air had affected their health in one way or another. The four main impacts include causing respiratory disease, shortness of breath, uncomfortable feelings, and inconveniences from dust and smog.

**Table 4:** Response to impacts of polluted air.

Impacts	Number	Percentages
Respiratory disease	163	56.2%
Dust, smog	158	54.5%
Shortness of breath	140	48.3%
Uncomfortable feelings	136	46.9%
Not impacted/Normal	23	7.9%
Other impacts	8	2.8%

*Notes*: Interviewees can choose multiple impacts.

The second part of the data, which studies prevention to air pollution and suggested solutions to reduce air pollution, consists of three descriptive tables. Tables 5 and 6 present preventative ways to air pollution and the current level of air protection activities. About half of the interviewed residents choose to close doors and use different kinds of masks to avoid air pollution. A fewer number of interviewees bought air purifiers, stayed at home or traveled to other provinces on the weekends to get out of polluted air. More extreme measures, such as resettling down in new cities or foreign countries with less polluted air, are less favorable among interviewees. Regarding air information, only a small number of respondents know where to get up-to-date information on air quality. The limited information issue stresses the necessity for air quality forecast, which most participants highly agreed.

**Table 5:** Respondent's preventative measures to air pollution and air protection level.

Dimensions	N	Mean	SD	SE	95% CI		Variable meaning and measurement
					Lower Bound	Upper Bound	
OwnedAirPurifier	290	0.20	0.40	0.02	0.15	0.25	Have an air purifier? Measured on a binary scale (1=yes; 0=no)
TravelToAvoid	290	0.19	0.39	0.02	0.14	0.24	Travel on weekends more often? Measured on a binary scale (1=yes; 0=no)
HomeToAvoid	290	0.42	0.51	0.03	0.37	0.48	Stay at home more often? Measured on a binary scale (1=yes; 0=no)
MoveCity	290	0.09	0.28	0.02	0.05	0.12	Intention to move to another city. Measured on a binary scale (1=yes; 0=no)
MoveCountry	290	0.06	0.24	0.01	0.03	0.09	Intention to move to another country. Measured on a binary scale (1=yes; 0=no)
UrgentToSolve	290	1.47	0.59	0.03	1.40	1.53	Urgent level to tackle air pollution problem. Measured on a four-point scale (1=very urgent; 2=urgent; 3=normal; 4=not urgent)
AirForcastNecessary	290	1.53	0.66	0.04	1.46	1.61	Necessity of air quality forecast. Measured on a four-point scale (1=very necessary; 2=necessary; 3=normal; 4=not necessary)
IsAwareInter	290	0.24	0.43	0.03	0.19	0.29	Be aware of international organizations that monitor air quality in Hanoi. Measured on a binary scale (1=yes; 0=no)
IsAwareVietnam	290	0.59	0.49	0.03	0.54	0.65	Be aware of Vietnamese organizations that monitor air quality. Measured on a binary scale (1=yes; 0=no)
AirProtectSatisfaction	284	2.56	0.89	0.05	2.46	2.67	Satisfaction level with air protection activities. Measured on a five-point scale (1=very dissatisfied; 2= dissatisfied; 3=normal; 4=satisfied; 5=very satisfied)
AirProtectCurrent	284	46.27	19.43	1.15	44.00	48.54	Evaluation of current air protection activities. Measured on a 0–100 scale
AirProtectWish	285	84.42	13.21	0.78	82.88	85.96	Wish of future level of air protection activities. Measured on a 0–100 scale

Notes: SD, SE, and CI stand for standard deviation, standard error, and confidence interval, respectively. For the more detailed information of dimensions, see the codebook at: http://dx.doi.org/10.17632/rbh7nksbtc.1.

**Table 6:** Preventative measures to handle air pollution.

Measures	Number	Percentages
Close doors	155	53.4%
Wear activated carbon masks	130	44.8%
Wear medical masks	108	37.2%
Other preventative measures	86	29.7%
No prevention	15	5.2%
Wear respirators	5	1.7%

*Notes*: Interviewees can choose multiple measures.

The last three rows in Table 5 present the respondents’ perspective on current and future air protection activities. The finding shows that more interviewed residents are dissatisfied with the current level than the number of respondents who feel satisfied. Overall, the current level is below the average point, and interviewees would like to increase air protection activities by approximately twofold in the future. Table 7 below suggests some solutions to achieve that desire. Industrial and construction activities seem to be the most significant problems as most respondents recommended limiting such activities by either reducing construction dust or relocating industrial factories out of the city. Further recommendations include the easily doable measure of vacuuming and cleaning the road regularly along with somewhat a harder to be implemented solution of reducing transportation.

**Table 7:** Suggested solutions to improve air quality.

Solutions	Number	Percentages
Reduce construction dust	210	72.4%
Relocate industrial factories out of the city	204	70.3%
Vacuum, clean the road regularly	187	64.5%
Reduce transportation	178	61.4%
Other measures	137	47.2%
Increase environment tax	102	35.2%
Relocate schools/universities to other cities/regions	98	33.8%

*Notes*: Interviewees can choose multiple solutions.

**Fig. 3:**
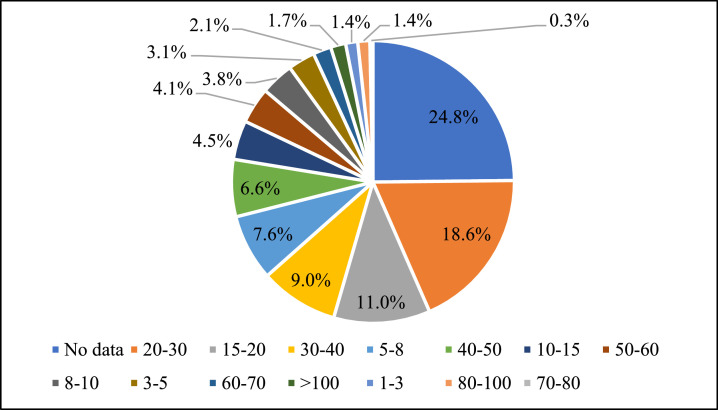
Regular income of the household of interviewees.

Notes: The income interval is measured in million Vietnamese Dong (VND). For instance, 5–8 stands for income from 5 million VND to 8 million VND.

The last part of the survey asks participants to provide personal information regarding age, gender, the highest education level, and members of their families (Table 8) alongside their income (Fig. 3). The number of interviewed residents whose age is above 30 accounts for 47.9% of total interviewees. There is a relatively high balance between the number of males and females who participated in the survey. Regarding education levels, 52.8% of participants have a bachelor's degree or above.

**Table 8:** Demographic information of interviewees.

Dimensions	N	Mean	SD	SE	95% CI		Variable meaning and measurement
					Lower Bound	Upper Bound	
AgeGroup	290	3.73	1.53	0.09	3.55	3.90	Age group of interviewees. Measured on a six-point scale (1= aged 10–18; 2=aged 19–30; 3= aged 31–40; 4=aged 41–50; 5=aged 51–60; 6=above 60)
Gender	289	0.57	0.50	0.03	0.51	0.63	Gender of interviewees. Measured on a binary scale (1=male; 0=female)
Education	286	3.05	1.20	0.07	2.91	3.19	Highest educational level attained (of the interviewees). Measured on a six-point scale (1= secondary school or below; 2=high school; 3= technical school/associate's degree; 4= bachelor's degree; 5=master's degree; 6=doctoral degree)
MainInHouse	290	0.56	0.50	0.03	0.50	0.62	Is the interviewee head of his/her household? Measured on a binary scale (1=yes; 0=no)
WorkingPlace	273	0.81	0.40	0.02	0.76	0.85	Working place of interviewees. Measured on a binary scale (1=indoor; 2=outdoor)
NumHouse	285	4.27	1.94	0.11	4.04	4.49	Number of family members
NumHouseMan	285	2.12	1.29	0.08	1.96	2.27	Number of male members in the family
NumHouseWoman	285	2.15	1.15	0.07	2.02	2.28	Number of female members in the family

Notes: SD, SE, and CI stand for standard deviation, standard error, and confidence interval, respectively. For the more detailed information of dimensions, see the codebook at: http://dx.doi.org/10.17632/rbh7nksbtc.1.

## 2 Experimental Design, Materials, Methods

**Experiment design and methods**. We selected Hanoi as a study area to gather data for several reasons. Hanoi is a fast-growing city in Vietnam [3]. Besides, this city belongs to the top largest and populous cities in Asia, while it is also considered one of the most polluted capital cities in the world [2]. To ensure the validity and reliability of the collected data, we followed three steps in designing our study [4]. First, we formed five focus groups whose members are sophomores at the National Economics University of Vietnam. This step is conducted to help the interviewers well understand the data-collecting procedures and help the focus groups iteratively refine the questionnaire. Second, we ran the pre-survey to test the survey and make it to arrive in the final version. The final version of the questionnaires, with 40 questions, was designed to obtain three sorts of desirable data. The first part of the questionnaires examines the interviewees’ understanding of air pollution and consists of 18 questions. The second part studies the preventive measures to either avoid or mitigate the negative impacts of polluted air on health and economics and contains 14 questions. The last section, with eight items, aims to collect the personal information and socio-economic features of respondents’ households.

Third, we conducted a survey using a stratified random sampling technique [4] and a face-to-face interview. We interviewed 302 respondents who live in seven out of eight suburban districts, including Ha Dong, Cau Giay, Tay Ho, Bac Tu Liem, Nam Tu Liem, Thanh Xuan, and Hoang Mai. It is noted that the suburban districts in Hanoi exclude the four former inner districts of Hoan Kiem, Hai Ba Trung, Dong Da, and Ba Dinh. The exclusion follows Decision No. 78-CP dated May 31, 1961, by the government that divided the inner areas and suburban areas of Hanoi city [5]. These four areas also have the highest land price adjustment coefficient regulated in the latest Decision No. 03/2020/QĐ-UBND dated March 2, 2020, by the Hanoi People's Committee [6]. During the interview process, the data-collecting team kept mutual interaction and continuous communication to correct issues or questions that arise during the survey.

**Sample size selection**. We followed previous environmental studies [7,8] in using the following equation to determine our sample size:

$$N=\frac{Z^{2}}{\sigma^{2}d^{2}}$$

Here, N is the (expected) sample size given a standard deviation σ, an allowable error d and the statistics under a specific confidence level Z.

Following [7], we set σ equal to 0.5. Due to limited human resources and financial constraints, we chose a confident level of 90% and a maximum allowable error level of 5%. According to Table 9 below, the sample size should be at least 271.

**Table 9:** Minimum sample size at various confidence and error levels.

Confidence level		80%	85%	90%	95%
**Error Level**	**1.0%**	4096	5148	6766	9604
	**2.0%**	1024	1296	1692	2401
	**3.0%**	456	576	752	1068
	**4.0%**	256	324	423	601
	**5.0%**	164	208	**271**	385
	**7.5%**	73	93	121	171

We intended to have at least 280–350 observations with 40–50 surveys per district to ensure our data's highest representativeness. After conducting the fieldwork, we collected a total of 302 surveys. We then eliminated highly implausible and incomplete observations and ultimately arrived in a valid sample size of 290.

Our study sample size is relatively close to the valid sample size of 330 in [9] that examined the public knowledge and awareness in waste management. It is also comparable to the city-level sample size of 249–262 in [7] that studied the willingness to pay to reduce air pollution in Beijing, Tianjin, and Hebei.

## Ethics Statement

The authors declare that this study is conducted with the willingness and approval of informed consent of all participants.

## Supplementary Material

Supplementary material associated with this article can be found in the online version at http://dx.doi.org/10.17632/rbh7nksbtc.1

## Declaration of Competing Interest

The authors declare that they have no known competing financial interests or personal relationships that could have appeared to influence the work reported in this paper.
